# Punishment and Reward Sensitivity in Risk-Taking as Potential Mechanisms Explaining the Relationships Between Childhood Callous-Unemotional Traits and Adolescent Substance Use in a Longitudinal Cohort Study Sample

**DOI:** 10.1007/s10802-024-01255-0

**Published:** 2024-10-23

**Authors:** Hanna Sakki, Michelle C. St Clair, Yiyun Shou, Jennifer L. Allen

**Affiliations:** 1 Department of Psychology, University of Bath, 10 West, Claverton Down, Bath, BA2 7AY UK; 2 Lloyd’s Register Foundation Institute for the Public Understanding of Risk, National University of Singapore, Singapore, 117602 Singapore; 3 Saw Swee Hock School of Public Health, National University of Singapore and National University Health System, Singapore, 117549 Singapore; 4 Research School of Psychology, Australian National University, Canberra, Australia

**Keywords:** Callous-unemotional traits, Substance use, Alcohol, Reward sensitivity, Punishment insensitivity, Risk-taking

## Abstract

Childhood callous-unemotional (CU) traits are associated with a neurocognitive response style of high reward and low punishment sensitivity, which may make these children particularly vulnerable to substance misuse. However, the mechanisms explaining the link between CU traits and substance use are poorly understood. This study investigated the mediating influences of reward and punishment sensitivity on the association between childhood CU traits and adolescent substance use. Using data from the UK Millennium Cohort Study, mediation analyses were conducted to investigate the potential indirect effects of age 14 reward and punishment sensitivity in risk-taking on the relationships between age 11 CU traits and alcohol, cannabis, and other illicit drug use at age 17. No direct effects of CU traits on substance use were found when accounting for gender, baseline alcohol use, poverty, emotional symptoms, conduct problems, hyperactivity, and verbal ability at age 11. Indirect effects of increased reward sensitivity on the relationship between CU traits and increased use were seen for alcohol, cannabis, and other drugs. There was a significant indirect effect of reduced punishment sensitivity on the relationship between CU traits and increased alcohol use, but not cannabis or other substance use. Findings suggest that reward and punishment sensitivity may have independent effects on decision-making processes contributing to adolescent substance use. Prevention and early intervention for substance use should consider modifying intervention strategies to fit the needs of adolescents with a callous interpersonal style and a neurocognitive profile characterized by a high drive for rewards and low risk aversion.

**Supplementary Information:**

The online version contains supplementary material available at 10.1007/s10802-024-01255-0.

Early substance use is associated with a range of adverse short- and long-term outcomes, including enduring substance use problems, poor educational attainment, career problems, and preventable disease and death (Gore et al., 2011; Room & Rehm, 2005). As well as the personal cost to the individual and their family, early substance use is pertinent to societal issues including the consequences of antisocial behavior and carries a substantial economic cost due to a high burden on education, legal, health and social care systems (Feinstein et al., 2012). However, experimenting with alcohol and other substances is considered a normative part of adolescence in western countries (Gray & Squeglia, 2018), and only a subset of young people transition to regular heavy use or to compulsive, uncontrolled use (Le Moal & Koob, 2007). Furthermore, although interventions targeting substance use in adolescence may be efficacious in the short term, longer-term outcomes are more variable (Hogue et al., 2014). It is therefore important to identify adolescents at high risk for adverse long-term outcomes and to develop personalized treatment programs based on the characteristics and context of the individual (Vincent et al., 2018).

Youth with antisocial behavior are a heterogeneous group that can be subtyped based on the temperament dimension of callous-unemotional (CU) traits, which are characterized by a lack of guilt, low empathy, restricted affect, and a lack of concern regarding performance (Frick et al., 2014). Children high in CU traits form a distinct subgroup of children with antisocial behavior, showing more severe, varied, and persistent antisocial behavior, a unique emotional, cognitive, and social-motivational profile, and poorer responses to intervention (Allen et al., 2020; Frick & Nigg, 2012). Children with high CU traits are at risk for the early onset of substance use and high CU traits are predictive of later substance abuse in adolescence (Thornton et al., 2019; Vincent et al., 2018; Wymbs et al., 2012). A meta-analysis found small significant associations between CU traits and frequency of substance use in adolescence (Winters et al., 2021), while a more recent meta-analysis found that these associations held even when controlling for age, gender, sample type, antisocial behavior and methodological factors related to study design and measurement of CU traits (Sakki et al., 2023).

The Reinforcement Sensitivity Theory (Gray, 1970; Gray & McNaughton, 2000) proposes that motivational systems underlying human behavior may be useful in identifying those with a neurocognitive predisposition for substance use beyond the normative developmental vulnerability. The Behavioral Inhibition System (BIS) relates to avoiding punishment (e.g., anxiety, risk assessment, threat anticipation), such that it responds to conditioned punishment cues with lowered motivation. The Behavioral Activation System (BAS) relates to reward sensitivity and approach behaviors (e.g., impulsivity, sensation-seeking, aggression), such that motivation is increased toward conditioned reward stimuli. Thus, the theory views risk-taking behaviors as being driven by both sensitivity to reward and an inability to inhibit behaviors likely to result in punishment, and previous research suggests that both constructs be investigated in the context of risk-taking (Kim-Spoon et al., 2016). In adolescent and young adult samples, higher BAS activation (higher reward sensitivity) has been associated with relatively less advantageous decision-making on a gambling task (Almy et al., 2018), impulsivity (Braddock et al., 2011), and a higher likelihood of lifetime substance use/experimentation (Van Leeuwen et al., 2011). In turn, BIS activation (higher punishment sensitivity) has been negatively associated with impulsivity (Braddock et al., 2011) and progression to regular cannabis use (Van Leeuwen et al., 2011).

Gambling tasks may be useful behavioral measures of punishment and reward sensitivity, as they collect data on different aspects of decision-making and the evaluation of the risks and benefits of obtaining rewards (Bentivegna et al., 2024). A recent scoping review on gambling tasks as measures of self-regulation and reward processing in general youth populations suggested that emotion dysregulation appeared to have a significant relationship with reward processing, although longitudinal research is needed to understand the direction of this association (Bentivegna et al., 2024). The Cambridge Gambling Task (CGT) measures decision-making in the context of risk rather than in uncertainty, and minimizes demands on learning, working memory and cognitive flexibility (Rogers et al., 1999). It provides six scores, and previous studies have selected Risk Taking and Risk Adjustment as key outcomes (Lewis et al., 2021; Murphy et al., 2001).

Risk Taking is measured by the average proportion of points risked per trial, and has been characterized as a measure of reward sensitivity in past studies using this task (with higher scores indicating higher reward sensitivity; Flouri et al., 2022; Lewis et al., 2021). In youth samples, it has been positively associated with externalizing problems (Flouri et al., 2022) male gender (Lewis et al., 2021), emotion dysregulation (Francesconi et al., 2023) and attention deficit hyperactivity disorder, especially when comorbid with conduct disorder (Groen et al., 2013). Conversely, Risk Taking has been negatively associated with internalizing problems (Flouri et al., 2022) and cognitive regulation (Francesconi et al., 2023).

Risk Adjustment is measured by the extent to which the participant adjusts their risk-taking depending on the proportion of boxes of their chosen color (higher scores indicate higher likelihood of response modulation when the probability of the outcome changes, and thus higher punishment sensitivity). In youth samples, it has been positively associated with intelligence (Flouri et al., 2019), and was higher in females (Lewis et al., 2021), and emotion dysregulation (Poon, 2018). Further, a large UK longitudinal community sample found low Risk Adjustment to mediate the relationship between emotion dysregulation in childhood and eating disorder symptoms in adolescence (Francesconi et al., 2023). Risk Adjustment has also been positively associated with bullying, suggesting that this is a measure of situational deliberation and risk modulation, rather than of impulsivity (Poon, 2016).

Children with elevated CU traits show insensitivity to punishment cues when accounting for confounders including hyperactivity, cognitive ability, and environmental factors (for reviews, see Byrd et al., 2014; Frick et al., 2014). There is behavioral and neuroimaging evidence for deficits in emotional arousal to punishment, hindering the development of associative learning for punishment cues, especially when a reward-oriented response is primed (Byrd et al., 2014; Murray et al., 2018). However, it is uncertain whether punishment insensitivity pertains specifically to youth with high CU traits or characterizes the broader group of youth with antisocial behavior (Fung et al., 2005). Further, some evidence suggests that CU traits are associated with a reward-dominant motivational style, although findings are somewhat inconsistent and may be influenced by trait anxiety (Byrd et al., 2014; Frick et al., 2014; Waller & Hicks, 2019).

Studies to date have mainly focused on the direct relationship between CU traits and substance abuse, with little research investigating mechanisms that may explain their association. A question that remains is whether risk-taking in youth with high CU traits may be driven by high reward sensitivity, low punishment sensitivity, or some combination of both (Byrd et al., 2014). Therefore, the current study investigated the mediating influences of age 14 reward and punishment sensitivity in risk-taking on the relationships between age 11 CU traits and age 17 substance use frequency in the Millennium Cohort Study (MCS), a prospective, population-based mixed-gender cohort study in the United Kingdom (UK). It was hypothesized that 1) CU traits would be positively associated with substance use (alcohol, cannabis, other illicit drugs), 2) CU traits would be positively associated with high reward sensitivity and low punishment sensitivity, and 3) the relationship between CU traits and later substance use would be mediated by high reward and low punishment sensitivity.

## Method

### Participants and Design

The MCS is an ongoing longitudinal study of children born in the UK between September 2000 and January 2002, representative of the UK population. Stratified cluster randomized sampling was conducted, with over-sampling of children living in disadvantaged areas and smaller nations of the UK, and of ethnic minority backgrounds (Connelly & Platt, 2014). The baseline sample contained 18,818 children and participants remain eligible for further assessment timepoints if they live in the UK at the time (Fitzsimons et al., 2020). Further information can be found on the MCS website (http://www.cls.ioe.ac.uk/millennium-cohort-study/).

A longitudinal design was used, analyzing data at 11, 14 and 17 years. For families with multiple participating siblings, only one child was included (cohort member 1). Families gave written informed consent to participate, and ethics approval was obtained for all MCS timepoints (full details provided in Ipsos MORI, 2017, 2019; Mostafa, 2014). The University of Bath Psychology Research Ethics Committee granted ethics approval for this secondary data analysis (PREC code: 20–099).

## Measures

### Callous-Unemotional Traits (age 11)

CU traits were measured using 4 items from the 24-item youth self-report version of the Inventory of Callous-Unemotional Traits (ICU; Frick, 2004), which comprises three subscales: Callousness, Uncaring and Unemotional traits. ICU items are rated on a 4-point scale from 0 (not at all true) to 3 (definitely true), with higher scores indicating higher CU traits. The four items included: “I care about how well I do at school” (Uncaring subscale), “I feel bad or guilty when I have done something wrong” (Callousness subscale), “I do not show my emotions to others” (Unemotional subscale), and “I am concerned about the feelings of others” (Uncaring subscale). These four ICU items were selected for inclusion in the MCS because they were the items that loaded the most consistently onto the CU traits factor in clinical and community samples (Frick et al., 2000). The original 24-item ICU total score and Callousness and Uncaring subscales have acceptable internal consistency (pooled α = 0.75 – 0.83) and good external validity with psychological and behavioral outcomes (pooled r = 0.29 – 0.35, medium effect sizes), while the Unemotional subfactor has shown lower, although acceptable, internal consistency (pooled α = 0.71) but poor external validity with psychological and behavioral outcomes (pooled r = 0.05, please see Cardinale & Marsh, 2020 for a review). This has led to calls for the Unemotional scale to be removed from the ICU (Hawes et al., 2014; Kimonis et al., 2008), although other researchers argue that CU traits comprise one overarching construct which should not be subdivdided, and advocate for the use of the ICU total score (Ray & Frick, 2020).

In the MCS sample, while the two Uncaring items and the Callousness item were significantly related (*ρ*s = 0.30-0.33, *p*s < 0.001), the Unemotional item showed negligible correlations with the other items (*ρ*s < 0.10, see supplementary file). To investigate this further, the sample was divided into two random halves, with exploratory factor analysis conducted on the construction half indicating that a 1-factor solution was the best fit for the data, and that the Unemotional item did not load onto this factor. Confirmatory factor analysis (CFA) results from the whole MCS sample indicated that the 3-item ICU had an excellent fit to the data. Likewise, when the CFA was repeated in the analytic sample, the 3-item ICU had an excellent fit (see supplementary file). Therefore, the three Uncaring and Callousness items were summed to form a CU traits score (score range 0–9) and the Unemotional item was not included, in accordance with the findings of the meta-analysis of Cardinale and Marsh (2020) and as recommended by Hawes et al. (2014) and Kimonis et al. (2008).

### Reward and Punishment Sensitivity in Risk-Taking (age 14)

MCS cognitive assessment battery included the Cambridge Gambling Task (CGT; Atkinson, 2015). There is no recommended normative data for the CGT, so scores are described in relation to the total MCS analytic sample (https://www.cambridgecognition.com/cantab/cognitive-tests/executive-function/cambridge-gambling-task-cgt/). The CGT was administered on a tablet in the participant’s home. In each trial, the participant was presented with ten red and blue boxes of varying ratios (9:1, 8:2, 7:3, 6:4) with a yellow token hidden behind one box. The aim was to win as many points as possible by choosing the correct color of the box hiding the yellow token (decision-making) and betting points on decisions (risk-taking). The participant started with 100 points and then decided on the proportion of points to gamble on each decision. Depending on the task phase, the proportion of points to bet started low (5% of total points), increasing over 4 two-second intervals, or high (95% of total points), decreasing over 4 two-second intervals. At the end of each trial, the participant won points for a correct decision or lost the equivalent number of points for an incorrect decision. These points were added to or subtracted from the total score. The ascending stage was presented first to all participants. The task assessor recorded observations of issues that may have affected administration or performance (e.g., technical problems, background noise, other disturbances). Details of variable derivation are described in the MCS research guide (Atkinson, 2015). In line with previous MCS cohort research using CGT data (Lewis et al., 2021), Risk Taking was used as an index of reward sensitivity, and Risk Adjustment was used as a measure of punishment sensitivity.

### Substance Use (age 17)

Substance use in the past 12 months was recorded in the young person self-completion questionnaire. Alcohol use was categorized on a seven-point scale (1: never, 7: 40 or more times, see supplementary file Table S1). Use of other substances were categorized on five-point scales (1: not taken in last year, 5: more than 10 times, see supplementary file). Cannabis was analyzed separately due to indications that different substances have different patterns of use and may therefore have effects on the brain (U.S. Department of Health and Human Services & Office of the Surgeon General, 2016). All other substances were collapsed into an overall ‘other illicit drugs’ category due to low endorsement. Alcohol use was treated as a continuous variable. Cannabis use and other illicit drug use were treated as binary yes/no variables due to bimodal data distributions.

### Covariates (age 11)

A range of covariates were used. Gender was included given evidence for gender differences in the severity and correlates of CU traits (Cardinale & Marsh, 2020). One item from the child self-completion questionnaire was used to control for baseline alcohol intake (“Have you ever had an alcoholic drink? That is more than a few sips”) as ﻿early substance use initiation is associated with later disordered use (Swendsen et al., 2012). The same question was included at age 14 for a sensitivity analysis. The emotional symptoms, conduct problems and hyperactivity scales of the parent-report Strengths and Difficulties Questionnaire (SDQ; Goodman, 1997) were used to account for co-occurring psychopathology. The SDQ is a 25-item screening questionnaire measuring different child psychological attributes with items scored on a three-point scale (0: not true, to 2: certainly true) and each scale containing five items. It has good reliability and validity (mean internal consistency α = 0.73, mean retest stability r = 0.62 at 4–6 months, high SDQ scores associated with a substantial increase in psychiatric risk, with odds ratios of about 15 for parent and teacher SDQ scales; Goodman, 2001). The Verbal Similarities subtest of the British Abilities Scales-2, (BAS-2 VS; Elliott, Smith, & McCulloch, 1997) was used to account for cognitive ability, associated with performance on the CGT in the MCS cohort (Flouri et al., 2019). It requires the child to say how sets of three words are similar to each other. The BAS-2 is standardized in a representative sample of UK children, has high test–retest reliability, and good validity as a measure of cognitive ability (Elliott et al., 1997). The task was administered according to the manual and age-standardized T-scores were used. Poverty is a risk factor for child psychopathology (Forrest et al., 2020) and risk/punishment sensitivity (Herzberg & Gunnar, 2020), so the OECD below 60% median poverty indicator variable was used. 

## Statistical Analyses

Data preparation was carried out in SPSS 26 (IBM Corp, 2019). All statistical analyses were weighted according to the MCS whole UK sampling weights, based on the latest time-point of data included in each analysis. These sampling weights account for both non-response and survey design factors at each time-point in the study (Fitzsimons et al, 2020; Jones & Ketende, 2010). Completed cases were included in the main analyses. To assess the nature and extent of possible non-response biases, descriptive statistics and statistical group comparisons were checked for any systematic differences between the analytic and non-analytic samples. A sensitivity analysis was conducted including only those participants reporting no alcohol use at age 14, to allow for a possible stronger causal interpretation of the results.

To inspect the possible influence of missing data, further sensitivity analyses were conducted using multiple imputation using chained equations (MICE) to account for missing data on the covariates (Silverwood et al., 2024; Sterne et al., 2009). Data was assumed to be missing at random and 20 datasets were imputed, using all variables included in the main analyses and auxiliary variables (age 11 CGT scores, age 14 parent-reported SDQ emotional symptoms, conduct problems and hyperactivity, and age 14 endorsement of alcohol, cannabis and other drug use, age 14 OECD below 60% median poverty indicator and sweep at MCS study entry, country at entry (England vs. other) sweeps 5–7 non-response weights). For each analysis, we started with a sample that had complete data on the predictor and replaced the missing data in covariates. We used Rubin’s combination rules to consolidate the obtained individual estimates into a single set of multiply imputed estimates (Rubin, 2004).

Mediation analyses were run in STATA version 16 (StataCorp, 2019). The multiple mediator model of Hayes (2022, pp. 164–165) was used to calculate the direct, indirect and total effects. This model assumes that the variables of interest have a linear relationship, that there is no multicollinearity and no spurious outliers, but correlation between the IV and DV is not a requirement (Hayes, 2022, p. 82). The z-scoring method described by Iacobucci (2012) allowing categorical variables in mediation was followed (see supplementary file). Logistic regression was used when the DV was cannabis or other illicit drug use. Linear regression was used for all other models in the mediation analyses. As punishment insensitivity and reward sensitivity were considered conceptually distinct and were not highly correlated (Table 2), they were tested simultaneously as parallel mediators. All covariates were included in all stages of the models. Two-tailed significance tests were conducted with the *p*-value set at 0.05 and 95% confidence intervals were calculated.

## Results

### Sample Characteristics

The analytic sample was *n* = 7,021. Table 1 shows the descriptive statistics for the total, analytic and non-analytic samples, and statistical comparisons between the analytic and non-analytic samples (please see supplementary file for data distribution graphs and tables of the independent and dependent variables, Figures S1-S2 and Tables S2-S3). ICU3 scores were significantly higher in the non-analytic than analytic sample, and significantly more frequent age 17 alcohol use was reported for the analytic group than the non-analytic sample. No group differences were seen for age 17 cannabis or other illicit drug use. Both reward sensitivity and punishment insensitivity scores were significantly higher in the non-analytic than the analytic sample. Significantly higher SDQ scores and lower verbal ability scores were reported for the non-analytic than the analytic sample. A significantly higher percentage of males, baseline alcohol use, and below 60% OECD median income households were reported in the non-analytic than the analytic sample.

**Table 1:** Descriptive Statistics and Preliminary Analyses of the Test Scores of the Analytic and Non-Analytic Samples

			Analytic sample		Comparison					
Continuous variables	*n*		95% CI	M	95% CI	*n*	M	95% *CI*	*F*	*p*
Predictor	ICU3 (0-9 scale)	2.19	7,021	2.05	2.00 – 2.10	5,449	2.35	2.28 – 2.42	47.19	< .001
Mediators	Punishment sensitivity (-1 – 1 scale)	0.96	7,021	1.05	1.01 – 1.08	3,831	0.84	0.80 – 0.88	68.26	< .001
	Reward sensitivity (0.05 – 0.95 scale)	0.52	7,021	0.52	0.51 – 0.52	3,831	0.53	0.52 – 0.54	8.64	.003
Outcome	Alcohol (1 – 7 scale)	3.67	7,021	3.89	3.80 – 3.98	2,923	3.34	3.17 – 3.51	31.93	< .001
Covariates	SDQ Emotion (0 -5 scale)	1.93	7,021	1.82	1.76 – 1.89	5,829	2.04	1.96 – 2.12	19.14	< .001
	SDQ Conduct (0 – 5 scale)	1.50	7,021	1.28	1.23 – 1.33	5,832	1.74	1.68 – 1.81	141.00	< .001
	SDQ Hyperactivity (0-5 scale)	3.26	7,021	2.96	2.88 – 3.05	5,805	3.58	3.49 – 3.67	115.40	< .001
	BAS-2 VS (T-score)	58.27	7,021	59.62	59.10 – 60.15	6,146	56.87	56.31 – 57.43	140.16	< .001
			Analytic Sample		Comparison					
Binary variables	*n*		95% CI	%	95% CI	*n*	%	95% *CI*	*F*	*p*
Outcome	Cannabis	27.4	7,021	28.7	26.8 – 30.7	2,932	25.3	21.7 – 29.3	2.52	.11
	Drugs	9.2	7,021	9.8	8.7 – 11.0	2,908	8.4	6.2 – 11.3	0.95	.33
Covariates	Gender (male)	51.6	7,021	48.5	47.1 – 50.0	6,445	54.6	53.1 – 56.0	39.46	< .001
	Baseline alcohol	13.2	7,021	12.4	11.4 – 13.5	5,793	14.0	12.8 – 15.2	4.49	.04
	Poverty (< 60% median)	26.1	7,021	19.2	16.9 – 21.6	6,447	32.9	30.4 – 35.6	137.45	< .001

Weighted estimates of means, confidence intervals and percentages are reported. Unweighted counts are reported. For comparisons between analytic and non-analytic sample, Wald F tests were calculated using appropriate sampling weights, F (1, 389). *n* = number of participants with available data; *M* = mean; *CI* = confidence interval; *ICU3* = sum score of 3 ICU items; *Alcohol* = age 17 12-month alcohol use frequency; *Cannabis* = age 17 12-month cannabis use endorsed; *Drugs* = age 17 12-month other illicit drug use endorsed; *Baseline alcohol* = have tried an alcoholic drink at age 11; *Poverty* = below 60% OECD median household income; *SDQ* = Strengths and Difficulties Questionnaire; *Emotion* = Emotional Symptoms; *Conduct* = Conduct Problems; *Hyperactivity* = Hyperactivity; *BAS-2 VS *= British Abilities Scale 2 Verbal Similarities

### Mediation Analyses

The univariate associations between the predictor, mediators, outcomes, and covariates are shown in Table 2. The ICU3 score showed a small significant positive correlation with cannabis use but no associations were seen with alcohol or other illicit drug use. All predictor, mediator and outcome variables showed small significant associations with most covariates.

**Table 2:** Zero Order Correlations Between Test Variables

	ICU3	Punishment sensitivity	Reward sensitivity	Alcohol	Cannabis	Drugs	Gender (male)	Baseline alcohol	Poverty	SDQ Emotion	SDQ Conduct	SDQ Hyperactivity	BAS-2 VS
ICU3													
Punishment sensitivity	-.07***												
Reward sensitivity	.12***	-.23***											
Alcohol	-.03	.11***	.02										
Cannabis^a, b^	.04*	.04**	.08***	.43***									
Drugs^a, b^	.03	.01	.06***	.33***									
Gender (male)^a, b^	.22***	.10***	.25***	.01									
Baseline alcohol^a, b^	.12***	-.01	.02	.13*									
Poverty^a, b^	.11***	-.17***	.07***	-.20***									
SDQ Emotion	.10***	-.09***	-.03	-.16***	-.06***	-.04*	-.05***	.02	.15***				
SDQ Conduct	.21***	-.15***	.09***	-.09***	.05*	.41	.08***	.07***	.24***	.37***			
SDQ Hyperactivity	.24***	-.14***	.10***	-.10***	.02	.02	.18***	.09***	.19***	.36***	.56***		
BAS-2 VS	-.14***	.17***	-.05**	.14***	.07***	.06***	.04*	-.03	-.22***	-.18***	-.17***	-.20***	

Weighted analyses conducted on analytic sample only (*n *= 6,845). *p < .05, **p < .01, ***p < .00. ^a^Point-biserial correlations calculated for associations between one binary and one continuous variable. ^b^no correlations presented for associations between binary variables. *ICU3* = sum score of 3 ICU items; *Alcohol* = age 17 12-month alcohol use frequency; *Cannabis* = age 17 12-month cannabis use; *Drugs* = age 17 12-month other illicit drug use; *Baseline* alcohol = have tried an alcoholic drink at age 11; *Poverty* = below 60% OECD median household income; *SDQ* = Strengths and Difficulties Questionnaire; *Emotion* = Emotional Symptoms; *Conduct* = Conduct Problems; *Hyperactivity* = Hyperactivity; *BAS-2 VS* = British Abilities Scale 2 Verbal Similarities

**Fig. 1:**
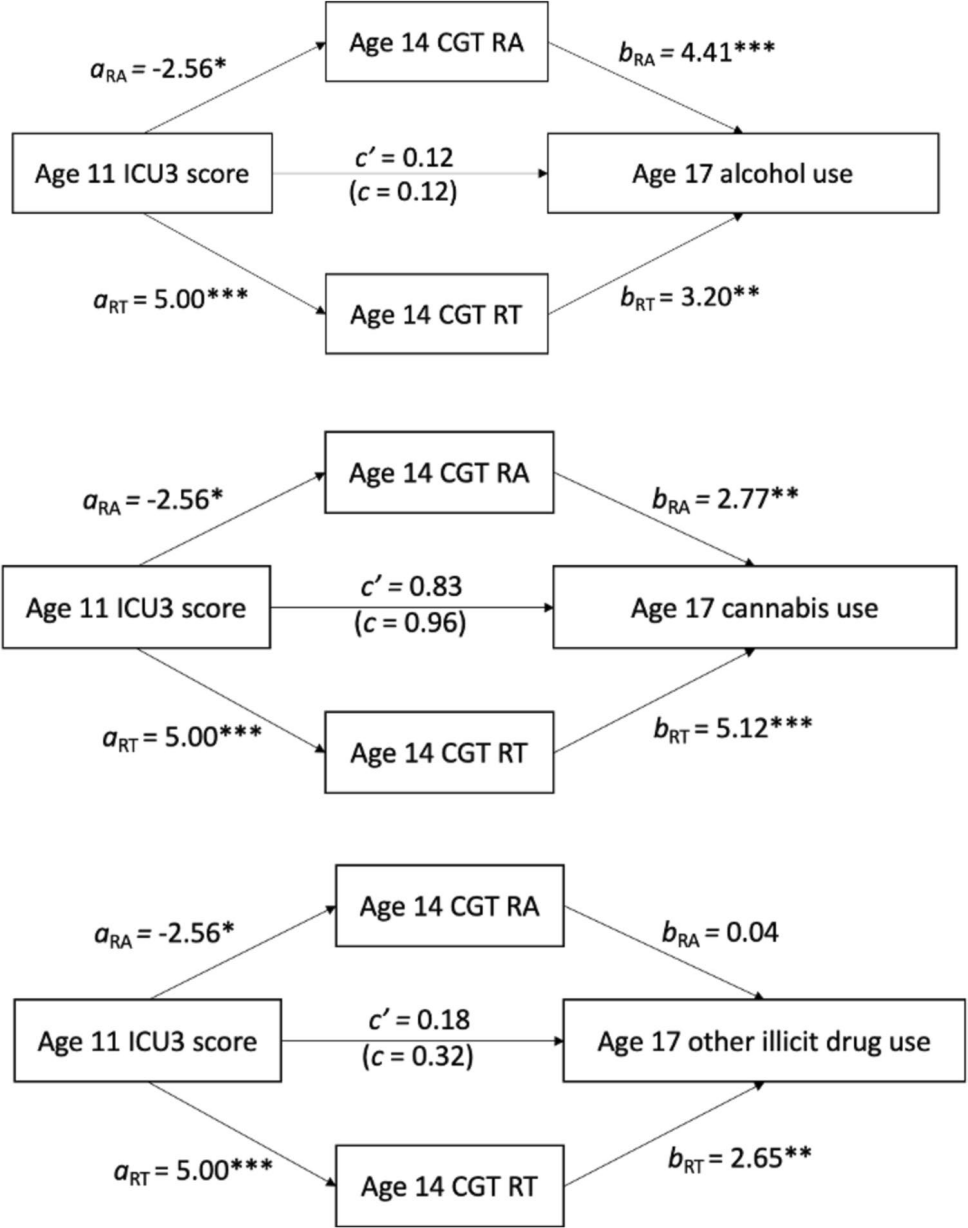
Path Models of Age 11 CU Traits on Age 17 Substance Use, mediated by Age 14 Reward and Punishment Sensitivity. Note. All effects are standardized z-scores, calculated according to Iacobucci, 2012 (see supplementary file). Covariates are included in each stage of each model, but only the primary variables of interest and the paths between them are presented for clarity. *p < .05, **p < .01, ***p < .001. CGT RA = Cambridge gambling task Risk Adjustment (punishment sensitivity); CGT RT = Cambridge gambling task Risk Taking (reward sensitivity)

Figure 1 shows the path models of ICU3 scores on substance use outcomes, mediated by reward and punishment sensitivity. Table 3 presents the regression coefficients of each stage of the mediation analyses, and Table 4 shows the indirect, direct, and total standardized effects of each model. Higher ICU3 scores were significantly predictive of lower punishment insensitivity (CGT risk adjustment) and higher reward sensitivity (CGT risk taking) adjusting for covariates (Fig. 1, Table 3).

**Table 3:** Regression Models for each Stage of the Mediation Analyses

Alcohol								
	ICU3 to substance use	ICU3 to Punishment sensitivity^b^			ICU3 to Punishment sensitivity & Reward sensitivity to substance use			
	*β*	95% CI	*β*	95% CI	*β*	95% CI	*β*	95% CI
Intercept	3.08***	2.66 – 3.50	0.51***	0.27 – 0.76	0.51***	0.47 – 0.54	2.63***	2.15 – 3.11
ICU3	0.00	-0.03 – 0.04	-0.02*	-0.04 – -0.01	0.01**	0.00 – 0.01	0.00	-0.03 – 0.04
Punishment sensitivity							0.14***	0.08 – 0.20
Reward sensitivity							0.75**	0.29 – 1.22
Gender (male)	-0.01	-0.12 – 0.11	0.24***	0.18 – 0.30	0.07***	0.06 – 0.08	-0.09	-0.21 – 0.03
Baseline alcohol	0.84***	0.62 – 1.05	0.04	-0.06 – 0.13	0.00	-0.02 – 0.01	0.83***	0.62 – 1.05
Poverty	-0.92***	-1.15 – -0.7	-0.28***	-0.37 – -0.20	0.02*	0.00 – 0.03	-0.9***	-1.12 – -0.67
SDQ emotion	-0.11***	-0.14 – -0.08	0.00	-0.02 – 0.02	0.00***	-0.01 – 0.00	-0.11***	-0.13 – -0.08
SDQ conduct	0.01	-0.04 – 0.06	-0.04**	-0.07 – -0.02	0.01*	0.00 – 0.01	0.02	-0.03 – 0.06
SDQ hyperactivity	-0.02	-0.05 – 0.01	-0.03***	-0.05 – -0.02	0.00	0.00 – 0.00	-0.02	-0.05 – 0.01
BAS-2 VS	0.02***	0.01 – 0.03	0.01***	0.01 – 0.02	0.00*	0.00 – 0.00	0.02***	0.01 – 0.02
Model effect (R^2^)	*F* (8, 382) = 31.28*** (.08)	*F* (8, 382) = 42.74*** (.07)			*F *(10, 380) =27.69*** (.09)			
Cannabis								
	ICU3 to substance use	ICU3 to punishment sensitivity^b^			ICU3 to punishment & reward sensitivity to substance use			
	OR	95% CI					OR	95% CI
Intercept	0.11***	0.06 – 0.19					0.05***	0.03 – 0.10
ICU3	1.02	0.90 – 1.07					1.02	0.97 – 1.07
Punishment sensitivity							1.14**	1.04 – 1.25
Reward sensitivity							3.44***	2.14 – 5.52
Gender (male)	1.24**	1.07 – 1.43					1.11	0.96 – 1.28
Baseline alcohol	2.32***	1.87 – 2.90					2.34***	1.88 – 2.91
Poverty	1.03	0.83 – 1.29					1.05	0.84 – 1.31
SDQ emotion	0.91***	0.88 – 0.95					0.92***	0.88 – 0.95
SDQ conduct	1.10**	1.03 – 1.18					1.10**	1.04 – 1.17
SDQ hyperactivity	1.00	0.97 – 1.04					1.01	0.97 – 1.04
BAS-2 VS	1.02***	1.01 – 1.03					1.02***	1.01 – 1.03
Model effect (R^2^)	*F* (8, 382) = 13.55*** (.03)^a^						*F* (10, 380) = 14.32*** (.03)^a^	
Other illicit drugs								
			ICU3 to punishment sensitivity^b^		ICU3 to reward sensitivity^b^		ICU3 to punishment sensitivity & reward sensitivity to substance use	
	OR	95% CI					OR	95% CI
Intercept	0.02***	0.01 – 0.04					0.01***	0.01 – 0.03
ICU3	1.01	0.94 – 1.09					1.01	0.93 – 1.09
Punishment sensitivity							1.00	0.87 – 1.16
Reward sensitivity							3.05**	1.33 – 6.98
Gender (male)	1.40**	1.09 – 1.81					1.30	1.00 – 1.71
Baseline alcohol	2.34***	1.70 – 3.22					2.35***	1.72 – 3.22
Poverty	0.76	0.50 – 1.16					0.74	0.48 – 1.15
SDQ emotion	0.92**	0.87 – 0.97					0.92**	0.88 – 0.97
SDQ conduct	1.14**	1.04 – 1.26					1.14**	1.03 – 1.25
SDQ hyperactivity	0.99	0.94 – 1.04					0.99	0.94 – 1.04
BAS-2 VS	1.02***	1.01 – 1.03					1.02***	1.01 – 1.03
Model effect (R^2^)	*F* (8, 382) = 11.48*** (.04)^a^						*F* (10, 380) = 9.62*** (.04)^a^	

*p < .05, **p < .01, ***p < .001. Odds ratios presented for logistic regression models. ^a^McKelvey & Zavoina’s pseudo R^2^ presented for logistic regressions. ^b^The stage 2 mediation model is the same for all substance use outcomes. *ICU3* = sum score of 3 ICU items; *Alcohol* = age 17 12-month alcohol use frequency; *Cannabis* = age 17 12-month cannabis use; *Drugs* = age 17 12-month other illicit drug use; *Baseline alcohol* = have tried an alcoholic drink at age 11; *Poverty* = below 60% OECD median household income; *SDQ* = Strengths and Difficulties Questionnaire; *Emotion* = Emotional Symptoms; *Conduct* = Conduct Problems; *Hyperactivity* = Hyperactivity; *BAS-2 VS* = British Abilities Scale 2 Verbal Similarities

A sensitivity analysis including only those participants reporting no alcohol use aged 14 was conducted to control for the influence of alcohol use on reinforcement sensitivity. This showed no major differences to the results of the mediation models including the full sample. See supplementary file Table S2 for details

**Table 4:** Indirect, Direct, and Total Effects of Reward and Punishment Sensitivity on Substance Use

	Cannabis						Other illicit drugs		
Path	*SE*_*z*_^a^	*z*_mediation_^b^	*z*^a^	*z*_mediation_^b^	*p*^b^	*z*^a^	*SE*_*z*_^a^	*z*_mediation_^b^	*p*^b^
Direct path between CU traits and substance use (c)	1.01		0.96			0.32	1.05		
Direct effect (*c’*)	1.01		0.83			0.18	1.02		
Specific indirect effect of punishment sensitivity (*a*_RA_*b_RA_)	5.19	-2.17	-7.07	-1.81	0.07	-0.10	2.74	-0.04	0.97
Specific indirect effect of reward sensitivity (*a*_RT_*b_RT_)	6.02	2.66	25.62	3.54	< .001	13.23	5.74	2.30	0.02
Total effect (*a*_RA_*b_RA_) + (*a*_RT_*b_RT_) + *c’*			19.38			13.31			

All models are controlled for the covariates. All effects are standardized z-scores. ^a^z score of the effect, ^b^z-test of the mediation effect. *CU* = callous-unemotional *SE* = Standard error

### Alcohol

There was no direct effect of age 11 ICU3 score on age 17 alcohol use frequency (Fig. 1, Table 3). Both punishment insensitivity and reward sensitivity showed significant specific indirect mediating effects on age 17 alcohol use frequency when controlling for the other mediator and all covariates in the model (Table 4).

### Cannabis

There was no direct effect of age 11 ICU3 score on age 17 likelihood of cannabis use (Fig. 1, Table 3). No specific indirect effect of punishment sensitivity was seen when controlling for reward sensitivity and covariates. There was a significant specific indirect effect of reward sensitivity when controlling for punishment insensitivity and covariates (Table 4).

### Other Illicit Drugs

There was no direct effect of age 11 ICU3 score on age 17 likelihood of other illicit use (Fig. 1, Table 3). No specific indirect effect of punishment sensitivity was seen when controlling for reward sensitivity and covariates, but a significant indirect effect of reward sensitivity was found when the other variables in the model were kept constant (Table 4).

### Sensitivity Analyses

There were no major differences seen between the main analyses reported and the sensitivity analyses using imputed data, or the sensitivity analysis including only those participants reporting no alcohol use at age 14 (see supplementary file Tables S4-S7 for full details).

## Discussion

This study is one of the first to consider potential mechanisms to explain the associations between CU traits and substance use in a large community sample of adolescents in the UK population, and found mediating paths between these constructs through both punishment and reward sensitivity in risk-taking. These results expand our understanding of the underlying mechanisms driving substance use in youth who are at risk for adverse substance use outcomes (Frick & Nigg, 2012). Higher CU traits at age 11 were a significant predictor of higher reward sensitivity in a gambling task at age 14, which was significantly predictive of higher alcohol at age 17, cannabis and other illicit drug use. Conversely, higher CU traits were a significant predictor of punishment insensitivity, which was a predictor of higher substance use, and this path only held for alcohol use frequency, not other substance use.

Overall, these results coincide with previous research reporting that elevated CU traits are associated with a distinct neurocognitive response style favoring risky decisions with higher rewards and with less attention to punishment cues (Nigg, 2006). It further suggests that although both punishment and reward sensitivity may play independent roles in decision-making processes contributing to adolescent substance use, this may be more driven by high reward sensitivity than low punishment sensitivity in a risk-taking context. This finding is somewhat contradictory to research reporting links between CU traits and punishment insensitivity (Moul et al., 2012; Murray et al., 2018). However, there is no clear consensus about the primary driver of risky behaviors in CU traits being high reward sensitivity, low punishment sensitivity or a combination of both (see Byrd et al., 2014) and questions have remained about influence of other factors on these relationships (e.g., anxiety, antisocial behavior; Fung et al., 2005; Waller & Hicks, 2019). As such, this study takes an important step forward in elucidating the paths between CU traits, reward and punishment sensitivity, and risk for substance use while controlling for known confounds.

Hypothesis 1, that CU traits at 11 years would be positively associated with substance use at age 17, was not supported. Contrary to previous research reporting small positive associations between CU traits and substance use (Sakki et al., 2023; Winters et al., 2021), no direct effects of CU traits were seen on substance use (i.e., the path from CU traits to substance use, not accounting for mediators) when accounting for covariates (namely gender, baseline alcohol use, poverty, emotional symptoms, conduct problems, hyperactivity, and verbal cognition). This difference may be related to study design factors, as for example the current study explicitly controlled for multiple covariates, which other studies with smaller sample sizes may have lacked the statistical power to do. The measurement of CU traits and substance use also differed across individual studies and most previous studies were cross-sectional (30 of 34 studies in a recent systematic review; Sakki et al., 2023) whereas the current study had a longitudinal design, and included a sensitivity analysis controlling for baseline alcohol intake (i.e., early alcohol intake not being the driving influence of later reward/punishment sensitivity in risk-taking), allowing for some consideration of the direction of effects.

Another factor which may explain the lack of a direct effect between CU traits and substance use in this study was the community sample, as high levels of CU traits endorsed at age 11 were low, with few children scoring at the higher end of the scale (0.4% of the analytic sample scoring the maximum 9 points). Although this is similar to other community samples reporting that only small numbers of children are high in CU traits (e.g., Kimonis et al., 2014; Rowe et al., 2010), this reduced variability may have limited the sensitivity of the analyses conducted.

As CU traits at age 11 were found to be a significant predictor of age 14 reward sensitivity and a significant negative predictor of age 14 punishment sensitivity in a gambling task, hypothesis 2 was supported. This aligns with previous research reporting that children with high CU traits show punishment insensitivity (Byrd et al., 2014) and high reward sensitivity (Roose et al., 2011). Hypothesis 3 that the relationship between age 11 CU traits and substance use aged 17 would be mediated by high reward and low punishment sensitivity in risk-taking was partially supported. Significant mediating effects of reward sensitivity were seen for all substance use models, suggesting that reward sensitivity has an indirect effect on substance use from CU traits, such that CU traits were a positive predictor of risk taking, which was a positive predictor of substance use. The indirect effect of punishment sensitivity was less clear, as although a significant mediating effect punishment sensitivity was found between CU traits and alcohol use frequency such that higher CU traits were related to lower punishment sensitivity, but higher punishment sensitivity was related to higher alcohol use, no significant mediating effects were seen in the cannabis and other illicit drugs models. Given the multitude of risk and protective factors related to adolescent substance use (Feinstein et al., 2012; Gore et al., 2011), further consideration of other factors or interactional effects that may contribute to substance use outcomes may be useful. For example, poor impulse control has been reported as a mediating factor between punishment sensitivity and substance use in youth with high CU traits and high anxiety (Waller & Hicks, 2019). Furthermore, the lack of a direct effect alongside the presence of significant mediation effects may suggest an opposing mediation effect of the paths to reward and punishment sensitivity in risk-taking from CU traits, and thus their combined effects may cancel each other out in the direct path to substance use (Hayes, 2022). This highlights the importance of considering third variable influences in the CU traits-substance use association.

Although the patterns of substance use in the MCS cohort at age 17 were comparable to a recent English population-based study (NHS Digital, 2019), it is possible that the very low rates of reported substance use other than alcohol may have impacted on the findings in this community sample. Notably, the alcohol use frequency model explained the largest amount of variability, and it is possible that the higher and more detailed measurement of alcohol in comparison to cannabis and other illicit drugs contributed to the comparatively better fit of this model. Similarly, it is possible that the non-significant effect of punishment sensitivity on cannabis and other illicit drugs may be related to the small numbers of participants endorsing any use at age 17.

These findings must be interpreted considering study limitations. Although the MCS cohort weighting adjusts for drop-out as well as stratified sampling, the non-response rates at each timepoint are higher for families from disadvantaged and ethnic minority backgrounds compared to advantaged areas (Ipsos MORI, 2019). In the current study, CU traits scores were significantly higher in the analytic than the non-analytic sample. Although this raises the possibility that children high in CU traits may not be fully represented, the effect size was very small, suggesting that this difference in the analytic and non-analytic samples may be due to the overpowered sample rather than clinically meaningful differences between these two samples. Further, sensitivity analyses using imputed covariate data to include a larger sample had concordant results to the main analyses. As the MCS collects a wealth of data across multiple domains, only brief measures could be included (Connelly & Platt, 2014). Only a subset of ICU items were available so these results are not standardized or validated and cannot directly be compared to normative data or other studies. Furthermore, because only three items from the questionnaire were included, this may have limited variability in the measure. However, items were selected because they loaded most consistently on the CU factor in community and clinical samples (Frick et al., 2000), and it is the quality, rather than quantity of items that matters most for construct validity (Heene et al., 2014; Ziegler et al., 2014). The 3-item ICU scale had an excellent fit to the data in the whole sample and the analytic sample, and showed significant associations in the expected direction with male gender, baseline alcohol use, conduct problems, hyperactivity, reward and punishment in risk-taking. The correlations between ICU3 scores and the SDQ conduct problems and hyperactivity scales (*r*s = 0.21, 0.24, respectively) were similar to the correlation between total ICU scores and externalizing symptoms in a study of UK school children aged 11 to 14 years (*r* = 0.21; Bird et al., 2019).

Both predictor and outcome variables were self-reported, raising the possibility of shared method variance. However, factors that lessen the risk of this having an undue influence on study findings include the temporal separation of self-report measures, and the assessment of mediators and covariates using behavioral tasks and parent-report (Podsakoff et al., 2012). Finally, information about alcohol use but not cannabis or illicit drug use was available at baseline, thus alcohol use was used as a proxy for these factors. Alcohol use is a strong predictor of both co-occurring and later cannabis and illicit drug use (Patton et al., 2007), and it is rare for adolescent cannabis and illicit drug use to not be preceded by alcohol use (Kosterman et al., 2000). However, ideally future longitudinal studies would collect information across all three substance use categories at baseline. From a theoretical perspective, it is prudent to note the non-significant correlation between age 11 CU traits and age 17 alcohol and drug use, as although the Hayes mediation model makes no assumptions between the presence of a correlation between the IV and DV for mediation analysis to be justified, some alternative mediation models do (Agler & De Boeck, 2017). As such, further research is needed to better understand these relationships. One limitation of research in this field is that reward and punishment sensitivity have been assessed using different measures/methods and thus conceptualized slightly differently across previous studies examining links with CU traits (Byrd et al., 2014), which makes contextualizing the current findings somewhat challenging, and highlights the need for conceptual clarity going forwards.

This study suggests the presence of an indirect effect of CU traits at age 11 through reward sensitivity in risk-taking aged 14, on age 17 self-reported substance use. Notably the overall regression models were all significant, supporting the hypothesized associations between covariates (gender, baseline alcohol use, poverty, internalizing and externalizing symptoms, and verbal ability) and outcomes. The results were less clear on the influence of punishment sensitivity (which had a significant mediating effect on alcohol use, but not other substances), which should be further investigated. Further, as the CU measure included in the MSC dataset only features a subset of the items in the longer validated ICU and has no normative data, it is not known whether the scores in this community sample would reach levels of clinical concern.

Longer-term follow-up of the MCS cohort at later timepoints would be valuable, as peak use of addictive drugs occurs during early adulthood (Kuhn, 2015). It would also be useful to specifically consider an index of problematic substance use (e.g., binge drinking or negative consequences of substance use). Further investigating the influence of other factors reported to play a role in the relationship between reinforcement sensitivity and substance use, such as inhibitory control (Kim-Spoon et al., 2016), negative affectivity (Rádosi et al., 2021), or anxiety (Waller & Hicks, 2019), may also be valuable to consider.

Finally, investigating youth in a clinical/forensic setting or those identified as high on CU traits based on newly available norms (Kemp et al., 2019), might help to better understand whether the findings from this community sample could be applied to those at clinical risk, and to inform interventions. The current findings suggest that taking an intensive reward-based approach to encourage the substitution of healthy alternative activities may help reduce substance use for this high-risk subgroup of children with antisocial behavior. Further, in adult male offenders with psychopathy, cognitive remediation treatment specifically aimed at improving attention to and integration of contextual cues in decision-making resulted in improved task performance above and beyond behavioral inhibition training (Baskin-Sommers et al., 2015). A similar approach might be useful in targeting attention to aversive cues and punishment sensitivity to reduce alcohol intake in youth with CU traits.

## Supplementary information

Below is the link to the electronic supplementary material.

ESM 1 (DOCX 110 KB)

## Data Availability

MCS is deposited with the UK Data Service at the University of Essex.
