# Supplementary material for: Punishment and Reward Sensitivity in Risk-Taking as Potential Mechanisms Explaining the Relationships Between Childhood Callous-Unemotional Traits and Adolescent Substance Use in a Longitudinal Cohort Study Sample
Source: Res Child Adolesc Psychopathol. 2024 Oct 23;53(1):1–15. doi: 10.1007/s10802-024-01255-0 (PMC11761481; doi:10.1007/s10802-024-01255-0)
Supplement: Supplementary file 1 — (DOCX 110 KB) [file 10802_2024_1255_MOESM1_ESM.docx]

## Supplementary file

## Additional methodological information

**Figure S1**

*Distribution of ICU3 Scores (Age 11)*

**Table S1**

*Substance Use Items Selected for this Study*

| **Question** | **Categorization** |
| --- | --- |
| Alcohol: How many times have you had an alcoholic drink in the last 12 months? | 1: Never  2: 1-2 times  3: 3-5 times  4: 6-9 times  5: 10-19 times  6: 20-39 times  7: 40 or more times |
| Drugs: In the past year how many times have you taken [*insert drug name*]? | 1: Not taken in last year  2: Once or twice  3: Three or four times  4: Five to ten times  5: More than ten times |
| *Note*. Drugs included cannabis, cocaine, acid/LSD, ecstasy, heroin, crack, speed/amphetamines, methamphetamine, semeron, ketamine, mephedrone, and psychoactive substances. | |

**Figure S2**

*Distribution of Alcohol Use Endorsed in the Past 12 Months (Age 17)*

**Table S2**

*Distribution of Cannabis use Endorsed in the Past 12 Months (Age 17)*

|  | No | Yes | Total |
| --- | --- | --- | --- |
| Excluded from final sample | 2,356 | 708 | 3,064 |
| Included in final sample | 5,166 | 1,830 | 6,996 |
| Total | 7,522 | 2,538 | 10,060 |

**Table S3**

*Distribution of Other Drug Use Endorsed in the Past 12 Months (Age 17)*

|  | No | Yes | Total |
| --- | --- | --- | --- |
| Excluded from final sample | 2,819 | 213 | 3,032 |
| Included in final sample | 6,343 | 607 | 6,950 |
| Total | 9,162 | 820 | 9,982 |

**ICU factor analysis details**

In the MCS sample, two-tailed Spearman rank correlations indicated that the two Uncaring items and the Callousness item were significantly related to one another (*ρ*s = .30-.33, *p*s < .001 for the total and analytic samples). In the total sample, whilst the Unemotional item was statistically significantly associated with the first Uncaring item ‘I care about how well I do at school’ and the Callousness item ‘I am concerned about the feelings of others’, the correlations were negligible (*ρ*s = .01 and .03, *p*s < .05 and .001). There was no correlation between the Unemotional and the second Uncaring item ‘I feel bad or guilty when I have done something wrong’ (*p* > .05).

To investigate the relationships between items further the sample was split into two halves at random, with exploratory factor analysis (EFA) conducted on the construction half of the sample, followed by confirmatory factor analysis (CFA) on the full MCS sample and the analytic sample of the current study.

The EFA was conducted to explore the factor structure. As the ICU items only had 4 response categories, we used the robust weighted least squares with mean and variance adjustment (WLSMV) estimator to minimise estimation bias. A one-factor model was identified with the first eigenvalue being 2.212. The remaining factors all had eigenvalues less than 1 (.466 - .826). However, while the two Uncaring and the Callousness items all loaded onto this factor (loadings ranging from 0.702 - 0.733, 49.28 – 53.73% of the variance being explained by the factor), the Unemotional item did not (loading = 0.369, 13.61% of its variance being explained). As the unemotional item had low correlations with other three items and was poorly predicted by the common factor, we removed the item from our final ICU model.

We then conducted CFA with WLSMV estimation in whole MCS sample separately for the 3-item CU traits scale. The main fit indices used to compare different models included: root-mean-square error of approximation (RMSEA ≤ 0.08 indicates an acceptable model fit), Tucker-Lewis index (TLI ≥. 90 indicates an acceptable model fit), comparative fit index (CFI ≥. 90 indicates an acceptable model fit), and standardized root-mean-square (SRMR values lower than .10 indicate acceptable fit). The 3-item ICU had an excellent fit to the data, CFI = 1.00, TLI = 1.00, SRMR = .00, RMSEA = .00. As a final check, CFA was repeated with only the analytic sample of this study. Again, the 3-item ICU had an excellent fit, CFI = 1.00, TLI = 1.00, SRMR = .00, RMSEA = .00. Therefore, the three Uncaring and Callousness items were summed to form a total CU traits score (range 0-9), and this 3-item CU traits total score was used in the main analyses.

**Details of the Iacobucci 2012 method to run mediation models with categorical and continuous variables**

1. A regression of the direct effect of ICU3 score on substance use outcome was fitted (*Y* = *b1* + *cX* ; path c).
2. Regression models of the effects of ICU3 score on risk taking and risk adjustment were fitted (*M*_risk taking_ = *b2* + a_RT_*X*; path a_RT_, and *M*_RA_= *b2* + a_RA_ *X*; path a_RA_).
3. A regression model was fitted to estimate the effects of RT and RA on substance use outcome, accounting for ICU3 score (*Y*=*b3* + *c′X* + *bM_RT_* +*bM*_RA_; paths b_RT ,_ b_RA_ and c’).
4. The parameter estimates and standard errors (SE) of paths a b_RT_ and b_RA_ were converted into z-scores.
5. Z-tests were calculated to evaluate the presence of significant mediation effects according to Hayes (2022).

**Table S4**

*Sensitivity analyses with only those reporting no alcohol use age 14 (n=6,345)*

| **Alcohol** | | | | | | | | |
| --- | --- | --- | --- | --- | --- | --- | --- | --- |
|  | ICU3 - > substance use | | ICU3 -> Risk Adjustment ^b^ | | ICU3 -> Risk Taking ^b^ | | ICU3 -> Risk Adjustment & Risk Taking -> substance use | |
|  | *β* | 95% CI | *β* | 95% CI | *β* | 95% CI | *β* | 95% CI |
| Intercept | 2.59*** | 2.07 – 3.10 | 0.70*** | 0.34 – 1.06 | 0.49*** | 0.45 – 0.53 | 2.24*** | 1.67 – 2.82 |
| ICU3 | 0.02 | -0.03 – 0.06 | -0.04*** | -0.06 – -0.02 | 0.00* | 0.00 – 0.01 | 0.02 | -0.03 – 0.07 |
| CGT Risk Adjustment |  |  |  |  |  |  | 0.15*** | 0.08 – 0.22 |
| CGT Risk Taking |  |  |  |  |  |  | 0.48 | -0.05 – 1.02 |
| Gender (male) | -0.03 | -0.18 – 0.11 | 0.24*** | 0.16 – 0.32 | 0.08*** | 0.06 – 0.09 | -0.10 | -0.25 – 0.04 |
| Baseline alcohol | 0.65*** | 0.37 – 0.94 | 0.08 | -0.05 – 0.22 | -0.02 | -0.04 – 0.00 | 0.65*** | 0.36 – 0.93 |
| Poverty | -0.90*** | -1.17 – -0.64 | -0.29*** | -0.40 – -0.18 | 0.03*** | 0.01 – 0.04 | -0.9*** | -1.14 – -0.61 |
| SDQ Emotion | -0.08*** | -0.12 – -0.05 | 0.00 | -0.02 – 0.02 | -0.01*** | -0.01 – -0.00 | -0.08*** | -0.12 – -0.05 |
| SDQ Conduct | 0.03 | -0.03 – 0.09 | -0.04 | -0.07 – -0.01 | 0.01* | -0.00 – 0.01 | 0.03 | -0.03 – 0.09 |
| SDQ Hyperactivity | -0.05** | -0.09 – -0.02 | -0.04*** | -0.06 – -0.19 | 0.00 | -0.00 – 0.00 | -0.05** | -0.08 – 0.02 |
| BAS-2 VS | 0.02*** | 0.01 – 0.02 | 0.01*** | 0.01 – 0.02 | 0.00 | -0.00 – 0.00 | 0.01*** | 0.01 – 0.02 |
| Model effect (R^2^) | *F* (8, 382) = 18.12*** (.08) | | *F* (8, 382) = 23.15*** (.08) | | *F* (8, 382) = 30.83*** (.10) | | *F* (10, 380) = 16.38*** (.09) | |
| **Cannabis** | | | | | | | | |
|  | ICU3 - > substance use | | ICU3 -> Risk Adjustment ^b^ | | ICU3 -> Risk Taking ^b^ | | ICU3 -> Risk Adjustment & Risk Taking -> substance use | |
|  | OR | 95% CI |  |  |  |  | OR | 95% CI |
| Intercept | 0.05*** | 0.02 – 0.13 |  |  |  |  | 0.02*** | 0.00 – 0.06 |
| ICU3 | 1.02 | 0.96 – 1.02 |  |  |  |  | 1.03 | 0.96 – 1.10 |
| CGT Risk Adjustment |  |  |  |  |  |  | 1.25** | 1.09 – 1.42 |
| CGT Risk Taking |  |  |  |  |  |  | 4.19** | 1.85 – 9.51 |
| Gender (male) | 1.40** | 1.10 – 1.77 |  |  |  |  | 1.19 | 0.93 – 1.53 |
| Baseline alcohol | 2.39*** | 1.58 – 3.61 |  |  |  |  | 2.42*** | 1.61 – 3.62 |
| Poverty | 0.97 | 0.64 – 1.47 |  |  |  |  | 0.99 | 0.65 – 1.50 |
| SDQ Emotion | 0.99** | 0.82 – 0.95 |  |  |  |  | 0.89** | 0.83 – 0.95 |
| SDQ Conduct | 1.15* | 1.04 – 1.29 |  |  |  |  | 1.16** | 1.04 – 1.28 |
| SDQ Hyperactivity | 0.98 | 0.92 – 1.04 |  |  |  |  | 0.99 | 0.93 – 1.05 |
| BAS-2 VS | 1.02* | 1.01 – 1.03 |  |  |  |  | 1.02* | 1.00 – 1.03 |
| Model effect (R^2^) | *F* (8, 382) = 6.53*** (.04) ^a^ | |  | |  | | *F* (10, 380) = 6.74*** (.05) ^a^ | |
| **Other illicit drugs** | | | | | | | | |
|  | ICU3 - > substance use | | ICU3 -> Risk Adjustment ^b^ | | ICU3 -> Risk Taking ^b^ | | ICU3 -> Risk Adjustment & Risk Taking -> substance use | |
|  | OR | 95% CI |  |  |  |  | OR | 95% CI |
| Intercept | 0.01*** | 0.01 – 0.02 |  |  |  |  | 0.02*** | 0.00 – 0.01 |
| ICU3 | 1.13 | 0.98 – 1.30 |  |  |  |  | 1.12 | 0.97 – 1.30 |
| CGT Risk Adjustment |  |  |  |  |  |  | 1.16 | 0.93 – 1.46 |
| CGT Risk Taking |  |  |  |  |  |  | 7.17* | 1.30 – 39.58 |
| Gender (male) | 2.32** | 1.30 – 4.16 |  |  |  |  | 1.95* | 1.04 – 3.64 |
| Baseline alcohol | 1.22 | 0.57 – 2.61 |  |  |  |  | 1.24 | 0.58– 2.64 |
| Poverty | 1.00 | 0.27 – 3.64 |  |  |  |  | 0.98 | 0.28 – 3.49 |
| SDQ Emotion | 0.85* | 0.73 – 0.98 |  |  |  |  | 0.86* | 0.74 – 0.99 |
| SDQ Conduct | 1.08 | 0.90 – 1.28 |  |  |  |  | 1.07 | 0.90 – 1.28 |
| SDQ Hyperactivity | 0.88* | 0.78 – 1.00 |  |  |  |  | 0.88* | 0.78 – 1.00 |
| BAS-2 VS | 1.02* | 1.00 – 1.04 |  |  |  |  | 1.02* | 1.00 – 1.04 |
| Model effect (R^2^) | *F* (8, 382) = 5.70.48*** (.09) ^a^ | |  | |  | | *F* (10, 380) = 5.72*** (.09) ^a^ | |
| Note. * p < .05, ** p < .01, *** p < .001 Odds ratios presented for logistic regression models. a McKelvey & Zavoina’s pseudo R2 presented for logistic regressions. b The stage 2 mediation model is the same for all substance use outcomes. ICU3 = sum score of 3 ICU items; CGT = Cambridge gambling task Risk Adjustment (punishment sensitivity) Risk Taking (reward sensitivity); Alcohol = age 17 12-month alcohol use frequency; Cannabis = age 17 12-month cannabis use endorsed; Drugs = age 17 12-month other illicit drug use endorsed; Baseline alcohol = have tried an alcoholic drink at age 11; Poverty = below 60% OECD median household income; SDQ = Strengths and Difficulties Questionnaire; Emotion = Emotional Symptoms; Conduct = Conduct Problems; Hyperactivity = Hyperactivity; BAS-2 VS = British Abilities Scale 2 Verbal Similarities.  Although the sensitivity analysis excludes those reporting alcohol use aged 14, baseline alcohol use included in the sensitivity analysis model to keep it standard with the main analysis.  When comparing this to the main analysis the main differences are that in the sensitivity analysis, risk taking is a non-significant variable for alcohol use in stage 3, SDQ hyperactivity is a non-significant predictor in stage 1 and stage 3 for the alcohol and other illicit drugs models, and SDQ hyperactivity is a significant predictor in the other illicit drug use model in stage 1 and 3. In general, the sensitivity analysis shows larger confidence intervals and p-values, as would be expected with a smaller sample. | | | | | | | | |

**Table S5**

*Sensitivity analyses using imputed data on covariates: Zero Order Correlations Between Test Variables*

|  | ICU3 | CGT Risk adjustment | CGT Risk taking | Alcohol | Cannabis | Drugs | Gender (male) | Baseline alcohol | Poverty | SDQ Emotion | SDQ Conduct | SDQ Hyperactivity | BAS-2 VS |
| --- | --- | --- | --- | --- | --- | --- | --- | --- | --- | --- | --- | --- | --- |
| ICU3 |  |  |  |  |  |  |  |  |  |  |  |  |  |
| CGT Risk Adjustment | -.07*** |  |  |  |  |  |  |  |  |  |  |  |  |
| CGT Risk Taking | .12*** | -.23*** |  |  |  |  |  |  |  |  |  |  |  |
| Alcohol | -.03 | .11*** | .03 |  |  |  |  |  |  |  |  |  |  |
| Cannabis ^a, b^ | .04* | .05** | .08*** | .43*** |  |  |  |  |  |  |  |  |  |
| Drugs ^a, b^ | .03 | .01 | .06*** | .33*** |  |  |  |  |  |  |  |  |  |
| Gender (male) ^a, b^ | .22*** | .10*** | .25*** | .01 |  |  |  |  |  |  |  |  |  |
| Baseline alcohol ^a, b^ | .11*** | .01 | .02 | .14* |  |  |  |  |  |  |  |  |  |
| Poverty ^a, b^ | .11*** | -.16*** | .07*** | -.22*** |  |  |  |  |  |  |  |  |  |
| SDQ Emotion | .10*** | -.10*** | -.02 | -.15*** | -.06*** | -.04* | -.05** | .02 | .15*** |  |  |  |  |
| SDQ Conduct | .21*** | -.15*** | .09*** | -.09*** | .04* | .04* | .08*** | .08** | .23*** | .38*** |  |  |  |
| SDQ Hyperactivity | .24*** | -.14*** | .10*** | -.10*** | .02 | .02 | .18*** | .09*** | .19*** | .36*** | .56*** |  |  |
| BAS-2 VS | -.15*** | .17*** | -.05** | .15*** | .07*** | .06*** | .04* | -.03 | -.22*** | -.18*** | -.17*** | -.20*** |  |
| *Note.* Weighted analyses conducted on non-imputed values only. * *p* < .05, ** *p* < .01, *** *p* < .001. ^a^ Point-biserial correlations calculated for associations between one binary and one continuous variable. ^b^ no correlations presented for associations between binary variables. ICU3 = sum score of 3 ICU items; CGT = Cambridge gambling task Risk Adjustment (punishment sensitivity) Risk Taking (reward sensitivity); Alcohol = age 17 12-month alcohol use frequency; Cannabis = age 17 12-month cannabis use; Drugs = age 17 12-month other illicit drug use; Baseline alcohol = have tried an alcoholic drink at age 11; Poverty = below 60% OECD median household income; SDQ = Strengths and Difficulties Questionnaire; Emotion = Emotional Symptoms; Conduct = Conduct Problems; Hyperactivity = Hyperactivity; BAS-2 VS = British Abilities Scale 2 Verbal Similarities. | | | | | | | | | | | | | |

**Table S6**

*Sensitivity analyses using imputed data on covariates: Regression Models for Each Stage of the Mediation Analyses*

| **Alcohol** | | | | | | | | |
| --- | --- | --- | --- | --- | --- | --- | --- | --- |
|  | ICU3 to substance use  (n = 8,797) | | ICU3 to Risk Adjustment ^a^  (n = 8,275) | | ICU3 to Risk Taking ^a^  (n = 8,275) | | ICU3 to Risk Adjustment & Risk Taking to substance use (n = 7,813) | |
|  | *β* | 95% CI | *β* | 95% CI | *β* | 95% CI | *β* | 95% CI |
| Intercept | 2.87*** | 2.40 – 3.33 | 0.43*** | 0.11 – 0.80 | 0.50*** | 0.44 – 0.55 | 2.64*** | 2.17 – 3.10 |
| ICU3 | -0.01 | -0.04 – 0.03 | -0.03** | -0.05 – -0.00 | 0.00** | 0.00 – 0.01 | 0.01 | -0.03 – 0.04 |
| CGT Risk Adjustment |  |  |  |  |  |  | 0.13*** | 0.08 – 0.19 |
| CGT Risk Taking |  |  |  |  |  |  | 0.80*** | 0.36 – 1.24 |
| Gender (male) | -0.02 | -0.13 – 0.10 | 0.24*** | 0.16 – 0.31 | 0.07*** | 0.06 – 0.08 | -0.10 | -0.21 – 0.02 |
| Baseline alcohol | 0.79*** | 0.59 – 0.98 | 0.02 | -0.10 – 0.14 | 0.00 | -0.02 – 0.01 | 0.84*** | 0.63 – 1.04 |
| Poverty | -0.95*** | -1.15 – -0.75 | -0.26*** | -0.42 – -0.16 | 0.02** | 0.00 – 0.04 | -1.01*** | -1.22 – -0.80 |
| SDQ Emotion | -0.11*** | -0.14 – -0.08 | 0.00 | -0.02 – 0.02 | 0.00*** | -0.01 – -0.00 | -0.10*** | -0.13 – -0.07 |
| SDQ Conduct | 0.03 | -0.02 – 0.08 | -0.04** | -0.06 – -0.02 | 0.00* | -0.00 – 0.01 | 0.02 | -0.03 – 0.09 |
| SDQ Hyperactivity | -0.02 | -0.05 – 0.00 | -0.03*** | -0.05 – -0.02 | 0.00 | -0.00 – 0.00 | -0.02 | -0.05 – 0.01 |
| BAS-2 VS | 0.02*** | 0.01 – 0.03 | 0.01*** | 0.01 – 0.02 | 0.00* | 0.00 – 0.00 | 0.02*** | 0.01 – 0.02 |
| Model effect | *F* (8, 385.2) = 33.50*** | | *F* (8, 386.8) = 49.42*** | | *F* (8, 386.8) = 46.49*** | | *F* (10, 386.9) = 31.78*** | |
| **Cannabis** | | | | | | | | |
|  | ICU3 to substance use  (n = 8,795) | | ICU3 to Risk Adjustment ^a^  (n = 8,275) | | ICU3 to Risk Taking ^a^  (n = 8,275) | | ICU3 to Risk Adjustment & Risk Taking to substance use (n = 7,810) | |
|  | OR | 95% CI |  |  |  |  | OR | 95% CI |
| Intercept | 0.05*** | 0.03 – 0.10 |  |  |  |  | 0.05*** | 0.03 – 0.09 |
| ICU3 | 1.03 | 0.99 – 1.07 |  |  |  |  | 1.02 | 0.98 – 1.06 |
| CGT Risk Adjustment |  |  |  |  |  |  | 1.16** | 1.06 – 1.26 |
| CGT Risk Taking |  |  |  |  |  |  | 3.61*** | 2.31 – 5.65 |
| Gender (male) | 1.29*** | 1.12 – 1.49 |  |  |  |  | 1.08 | 0.95 – 1.24 |
| Baseline alcohol | 2.26*** | 1.83 – 2.79 |  |  |  |  | 2.36** | 1.91 – 2.91 |
| Poverty | 1.15 | 0.89 – 1.49 |  |  |  |  | 0.96 | 0.78 – 1.18 |
| SDQ Emotion | 0.94** | 0.90 – 0.99 |  |  |  |  | 0.92*** | 0.88 – 0.96 |
| SDQ Conduct | 1.12*** | 1.06 – 1.19 |  |  |  |  | 1.10** | 1.03 – 1.16 |
| SDQ Hyperactivity | 1.00 | 0.97 – 1.03 |  |  |  |  | 1.01 | 0.98 – 1.04 |
| BAS-2 VS | 1.03*** | 1.02 – 1.04 |  |  |  |  | 1.02*** | 1.01 – 1.02 |
| Model effect | *F* (8, 386.2) = 16.25*** | |  | |  | | *F* (10, 386.9) = 16.51*** | |
| **Other illicit drugs** | | | | | | | | |
|  | ICU3 to substance use  (n = 8,737) | | ICU3 to Risk Adjustment ^a^  (n = 8,275) | | ICU3 to Risk Taking ^a^  (n = 8,275) | | ICU3 to Risk Adjustment & Risk Taking to substance use (n = 7,760) | |
|  | OR | 95% CI |  |  |  |  | OR | 95% CI |
| Intercept | 0.01*** | 0.00 – 0.04 |  |  |  |  | 0.01*** | 0.01 – 0.03 |
| ICU3 | 1.02 | 0.97 – 1.08 |  |  |  |  | 1.02 | 0.96 – 1.08 |
| CGT Risk Adjustment |  |  |  |  |  |  | 1.02 | 0.90 – 1.16 |
| CGT Risk Taking |  |  |  |  |  |  | 2.75** | 1.31 – 5.77 |
| Gender (male) | 1.45** | 1.16 – 1.82 |  |  |  |  | 1.34* | 1.05 – 1.71 |
| Baseline alcohol | 1.97*** | 1.41 – 2.75 |  |  |  |  | 2.33*** | 1.71 – 3.17 |
| Poverty | 0.85 | 0.54 – 1.36 |  |  |  |  | 0.69 | 0.45 – 1.05 |
| SDQ Emotion | 0.97 | 0.89 – 1.07 |  |  |  |  | 0.92** | 0.87 – 0.97 |
| SDQ Conduct | 1.17*** | 1.07 – 1.27 |  |  |  |  | 1.13** | 1.04 – 1.24 |
| SDQ Hyperactivity | 1.01 | 0.96 – 1.07 |  |  |  |  | 0.99 | 0.95 – 1.04 |
| BAS-2 VS | 1.03*** | 1.01 – 1.05 |  |  |  |  | 1.02*** | 1.01 – 1.03 |
| Model effect | *F* (8, 385.2) = 33.50*** | |  | |  | | *F* (10, 386.8) = 12.48*** | |
| *Note.* * *p* < .05, ** *p* < .01, *** *p* < .001. Odds ratios presented for logistic regression models. ^a^ The stage 2 mediation model is the same for all substance use outcomes. ICU3 = sum score of 3 ICU items; CGT = Cambridge gambling task; Alcohol = age 17 12-month alcohol use frequency; Cannabis = age 17 12-month cannabis use; Drugs = age 17 12-month other illicit drug use; Baseline alcohol = have tried an alcoholic drink at age 11; Poverty = below 60% OECD median household income; SDQ = Strengths and Difficulties Questionnaire; Emotion = Emotional Symptoms; Conduct = Conduct Problems; Hyperactivity = Hyperactivity; BAS-2 VS = British Abilities Scale 2 Verbal Similarities. | | | | | | | | |

**Table S7**

*Sensitivity analyses using imputed data on covariates: Indirect, Direct, and Total Effects of Reward and Punishment Sensitivity on Substance Use*

|  | **Alcohol** | | | | **Cannabis** | | | | **Other illicit drugs** | | | |
| --- | --- | --- | --- | --- | --- | --- | --- | --- | --- | --- | --- | --- |
| Path | *z* ^a^ | *SE_z_* ^a^ | *z*_mediation_ ^b^ | *p* ^b^ | *z* ^a^ | *SE_z_* ^a^ | *z*_mediation_ ^b^ | *p* ^b^ | *z* ^a^ | *SE_z_* ^a^ | *z*_mediation_ ^b^ | *p* ^b^ |
| Direct path between CU traits and substance use (c) | -0.29 | 1.04 |  |  | 1.43 | 1.74 |  |  | 0.88 | 1.33 |  |  |
| Direct effect (*c’*) | 0.42 | 1.08 |  |  | 1.17 | 1.54 |  |  | 0.60 | 1.16 |  |  |
| Specific indirect effect of CGT Risk Adjustment (*a*_RA_* *b*_RA_) | -14.38 | 5.66 | -2.54 | 0.01 | -10.67 | 4.74 | -2.25 | 0.02 | -1.055 | 3.29 | -0.32 | 0.75 |
| Specific indirect effect of CGT Risk Taking (*a*_RT_* *b*_RT_) | 11.88 | 4.99 | 2.38 | 0.02 | 18.63 | 6.66 | 2.81 | 0.01 | 8.80 | 4.36 | 2.02 | 0.04 |
| Total effect (*a*_RA_* *b*_RA_) + (*a*_RT_* *b*_RT_) + *c’* | -2.08 |  |  |  | 9.13 |  |  |  | 8.35 |  |  |  |
| *Note.* All models are controlled for the covariates. All effects are standardized z-scores. ^a^ z score of the effect, ^b^ z-test of the mediation effect. CU = callous-unemotional *SE* = Standard error; CGT = Cambridge gambling task Risk Adjustment (punishment sensitivity) Risk Taking (reward sensitivity). | | | | | | | | | | | | |
